# The role of Environmental Health in preventing antimicrobial resistance in low- and middle-income countries

**DOI:** 10.1186/s12199-021-01023-2

**Published:** 2021-10-05

**Authors:** David Musoke, Carol Namata, Grace Biyinzika Lubega, Filimin Niyongabo, Joviah Gonza, Kondwani Chidziwisano, Sarah Nalinya, Rebecca Nuwematsiko, Tracy Morse

**Affiliations:** 1 Department of Disease Control and Environmental Health, School of Public Health, College of Health Sciences, Makerere University, Kampala, Uganda; 2 Department of Environmental Health, University of Malawi, Polytechnic, Blantyre, Malawi; 3 Department of Civil and Environmental Engineering, University of Strathclyde, Glasgow, UK

**Keywords:** Environmental Health, Antimicrobial resistance, Wastewater, Solid waste, Water, Sanitation, Hygiene, Waste management, Food safety, One Health

## Abstract

Antimicrobial resistance (AMR) is increasingly becoming a threat to global public health, not least in low- and middle-income countries (LMICs) where it is contributing to longer treatment for illnesses, use of higher generation drugs, more expenditure on antimicrobials, and increased deaths attributed to what should be treatable diseases. Some of the known causes of AMR include misuse and overuse of antimicrobials in both humans and animals, unnecessary use of antimicrobials in animals as growth promoters, and lack of awareness among the public on how to protect antimicrobials. As a result, resistant organisms are circulating in the wider environment, and there is a need to consider the One Health approach to minimise the continuing development of AMR. Environmental Health, specifically water, sanitation and hygiene (WASH), waste management, and food hygiene and safety, are key components of One Health needed to prevent the spread of antimicrobial-resistant microorganisms particularly in LMICs and reduce the AMR threat to global public health. The key Environmental Health practices in the prevention of AMR include: (1) adequate WASH through access and consumption of safe water; suitable containment, treatment and disposal of human excreta and other wastewater including from health facilities; good personal hygiene practices such as washing hands with soap at critical times to prevent the spread of resistant microorganisms, and contraction of illnesses which may require antimicrobial treatment; (2) proper disposal of solid waste, including the disposal of unused and expired antimicrobials to prevent their unnecessary exposure to microorganisms in the environment; and (3) ensuring proper food hygiene and safety practices, such as sale and consumption of animal products in which adequate antimicrobial withdrawal periods have been observed, and growing vegetables on unpolluted soil. Environmental Health is therefore crucial in the prevention of infectious diseases that would require antimicrobials, reducing the spread of resistant organisms, and exposure to antimicrobial residues in LMICs. Working with other professionals in One Health, Environmental Health Practitioners have a key role in reducing the spread of AMR including health education and promotion, surveillance, enforcement of legislation, and research.

## Background

Antimicrobials have contributed to a reduction in infectious diseases, saving lives and increasing productivity. However, due to the extensive misuse of antimicrobials in humans and animals, there is an increasing development of antimicrobial resistance (AMR) which has escalated into a global health problem [1]. AMR leads to difficulty in controlling infections, increased treatment costs, and increased risk of death among patients. Globally, it is estimated that 700,000 people die annually due to drug-resistant infections [2], and the sustained increase in AMR may lead to 10 million deaths annually by 2050 worldwide [3]. In low- and middle-income countries (LMICs), infectious diseases account for more than half of the disease burden [4], and progress to reduce this high occurrence is now being hindered by increasing levels of resistant infections [5]. This concern is compounded by the lack of surveillance and up-to-date information regarding AMR among LMIC populations [6], which is needed for both the prevention and treatment of resistant infections.

To date, AMR prevention has largely focussed on antimicrobial stewardship, which promotes the appropriate use of antimicrobials in the management of patients. High-income countries have well established stewardship programmes but less so in LMICs [7]. Indeed, there remains a high level of improper use of antimicrobials in LMICs, including the unregulated sale of medicines, self-medication, non-adherence to treatment guidelines and prescriptions, and inappropriate use in animals and agriculture [6]. However, control of AMR is more than stewardship, as outlined in the WHO Global Action Plan [8]. This plan requires the development and support of National Action Plans across all countries using a One Health approach. This approach takes into consideration the complex interactions between humans, animals and the environment (including aquaculture) [9], recognising not only health but also the economic implications of widespread AMR. Recent evidence shows that AMR could cost low-income countries 5% of their gross domestic product and lead 28 million people into poverty by 2050 [10].

In the past two decades, researchers have been involved in understanding AMR mechanisms affecting clinical settings, and it is only recently that they have widened their focus to include the environment as a source of AMR [11]. Current evidence emphasises the need for a context appropriate One Health approach to address the growing development of AMR [12]. However, implementation of such an approach is extremely complex and requires a wide perspective. Environmental Health provides such an opportunity particularly in LMICs, with a focus on preventive health through a risk-based lens which encompasses the assessment and control of physical, chemical and biological factors in the environment that are likely to affect human health. As such, Environmental Health is well placed to understand the mechanisms for the mitigation and control of resistant organisms as they circulate within the wider population and environment. There is now significant evidence to suggest that improved Environmental Health factors such as water, sanitation and hygiene (WASH), waste management, as well as food hygiene and safety play a crucial role in the control of AMR [9, 13]. Understanding environmental pathways of AMR is vital for health professionals and communities to interrupt the development and spread of resistant organisms, including ingestion of antimicrobial residues. This commentary describes key Environmental Health factors and the role of the discipline in the prevention of AMR in LMICs (Table 1).

**Table 1:** Environmental Health factors and the role of Environmental Health Practitioners in prevention of antimicrobial resistance in low- and middle-income countries

Environmental Health factors in the prevention of AMR	Environmental Health Practitioners’ role in reducing the spread of AMR
1. Water, sanitation and hygiene - Access and consumption of safe water - Containment, treatment and disposal of human excreta and other wastewater including from health facilities - Personal hygiene such as washing hands with soap at critical times 2. Solid waste management, including disposal of unused and expired antimicrobials 3. Food hygiene and safety - Observance of antimicrobial withdrawal periods in animals - Growing vegetables on unpolluted soil	- Water sampling and analysis - Sanitary inspection of water sources - Inspection of premises including schools, markets, landing sites, and other institutions - Inspection of food, abattoirs and public eating places - Medical examination of food handlers - Health education and promotion - Surveillance - Enforcement of legislation - Research

### Water, sanitation, and hygiene

Nearly 827,000 deaths annually in LMICs are attributed to poor WASH conditions [14]. This represents 60% of the total diarrhoeal deaths, reported as the second leading cause of mortality in children under the age of five years, and accounting for over half a million deaths in this age group alone [14]. LMICs suffer a substantial burden of WASH-related diseases including cholera, dysentery, diarrhoea, hepatitis A, and typhoid that are habitually treated with antimicrobials [15]. However, most diarrhoeal diseases are caused by viruses and not bacteria, yet most people use antibiotics, often self-medicating, against these illnesses. The use of antimicrobials could be reduced by 60% if there was universal access to improved WASH services in LMICs [16].

Due to the over-consumption of antimicrobials by humans, excreta is known to be a major source of both antimicrobial agents and resistant genes [13]. As such, lack of sufficient sanitary facilities such as latrines and basic hygiene practices, particularly handwashing in LMICs, can lead to the spread of resistant organisms in the environment. The WHO estimates that globally, two billion people do not have basic sanitation facilities such as latrines, out of which 673 million practice open defaecation in street gutters, behind bushes, or into open water bodies [14]. This contributes to over 524 billion kilogrammes of human faecal biomass produced in LMICs, where open defaecation plays a significant role in releasing resistant organisms into the environment [15]. Even when faeces are contained, in many LMICs, the majority of the population use onsite sanitation facilities such as pit latrines and septic tanks. When human and animal excreta containing resistant microorganisms is disposed of indiscriminately, it is easily washed by rain into surface water sources such as rivers and lakes, and groundwater through seepage leading to the spread of resistant infectious diseases. Although wastewater treatment plants are deemed effective in eliminating many pathogens, evidence shows that they are not ordinarily designed for removal of antimicrobial residues or resistant organisms [17]. Therefore, discharge of treated wastewater into the environment also contaminates water bodies leading to the spread of resistant infections. This is of particular concern at institutional level, where high use of antimicrobials in hospitals has been shown to result in a large concentration of antimicrobial residues in effluent released into the environment, where it has been described as a key driver of AMR [18].

Consideration must also be given to the role of animal faeces, as this equates to 80% of the total faecal biomass in the world [19]. There is growing evidence of a high level of carriage of resistant organisms in livestock in LMICs, where animals are accommodated in people’s homes for security and other reasons [20]. Animals poorly absorb antibiotics in their guts, yet their waste does not usually undergo the secondary treatment human waste may be subject to, leading to the excretion of high concentrations of antibiotic residues in their faeces and urine. As a result, indiscriminate defaecation and urination by domesticated animals and livestock leads to a high number of antimicrobial-resistant genes being released into the environment including water bodies [13].

Taking into consideration the current status of WASH infrastructure and behaviours, LMIC environments are likely to remain heavily contaminated for the foreseeable future. As such, acceleration of WASH interventions (both infrastructure and behaviours) including proper handwashing with soap, clean play environments for children, animal containment, and clean water for drinking are critical for reducing both diarrhoeal diseases and the widespread distribution of resistant organisms and genes within the environment [15]. There is also a growing public health concern of resistant organisms from human and animal waste getting into water sources. If such water is not sufficiently treated before being used for drinking, washing and other domestic purposes, it exposes individuals to these resistant microorganisms [13]. Therefore, ensuring appropriate disposal of health facility effluent is not only vital for breaking the chain of transmission of pathogens between humans and the environment, but also impeding the spread of antimicrobial-resistant organisms.

Wastewater is known to be one of the common sources of a multitude of bacteria and other microbes such as *E. coli*, *Klebsiella* spp., *Shigella* spp., *Salmonella* spp., *Vibrio* spp., *Acinetobacter* spp., and *Enterococcus* spp. including those which are resistant to antimicrobials. The increased incidence of infections from such microorganisms leads to a high demand for the use of antimicrobials, which if not handled properly, contributes to the development of resistance. When wastewater is not sufficiently managed in the environment, it is believed that soil becomes a hotspot for transfer of the antimicrobial-resistant organisms to plants. This process occurs through transpiration and capillary action, providing a pathway of resistance through the food chain to both humans and animals. However, there is inadequate evidence to support human exposure to soil-born resistance [9]. Nonetheless, soil receives a large proportion of excreted antimicrobials from manure, human faeces, and sewage sludge as fertilisers. These antimicrobial residues are believed to be absorbed by food crops grown in manure applied soils [13]. For instance, wheat has been reported to take up chlortetracycline [13] which when consumed by humans creates pressure that enhances resistance of pathogenic organisms to antimicrobials.

### Solid waste management

While per capita waste generation is highest among high income countries, their improved solid waste management practices minimise health risks. However, in LMICs solid waste management remains an immense public health challenge, particularly in urban areas [21]. The rapid urbanisation of LMICs, which has led to overcrowding and growth of slums, contributes significantly to poor household solid waste management, alongside improper disposal from commercial businesses, industries, farms, clinics and hospital settings. This is particularly concerning when we consider that there is increased disposal of unused and expired antimicrobials together with other household waste in the environment [22]. This indiscriminate disposal of antimicrobials and general waste leads to contamination of various water sources including rivers and lakes. In addition, improper solid waste disposal in streets, rivers and drainage channels leads to flooding and accumulation of waste water. This creates favourable breeding places for mosquitoes which transmit infections such as malaria, dengue and yellow fever. Such conditions also favour the growth, survival and transmission of many organisms that cause infectious diseases such as cholera, diarrhoea, dysentery and respiratory complications [21]. These diseases account for high rates of morbidity in LMICs resulting in over use and reliance on antimicrobials [15]. Thus, the importance of solid waste management in the prevention of AMR cannot be overemphasised.

### Food hygiene and safety

Globally, it is estimated that unsafe food causes about 600 million cases of foodborne diseases and 420,000 deaths annually, resulting in the loss of 33 million healthy life years [23]. Immunocompromised individuals such as the elderly and children are most affected, with 40% of the foodborne disease burden occurring among children under the age of five years, leading to 125,000 deaths annually [23]. There is an increasing occurrence of foodborne illnesses in many LMICs, which continue to affect health and wellbeing of the population [24]. Besides health implications, foodborne illnesses hinder socio-economic development by harming economies, tourism and trade [25], and straining health care systems [23]. These concerns indicate that foodborne conditions are of significant public health importance as their high occurrence increases the need for use of antimicrobials during treatment.

Food from animals plays an important role in the spread of resistant microorganisms where food contamination can occur along the food production chain from the farm to consumption [25], including during slaughtering of animals. Food can contain resistant pathogens when the animal was infected, or when food was contaminated with unsafe fluids, flies, dirty fingers or utensils. In addition, the high volume of antimicrobials used among food-producing animals mainly for their growth and development as well as disease prevention contributes to the growth of resistant microorganisms. Another key concern in animal husbandry is the non-observance of adequate withdrawal periods which leads to accumulation of antimicrobial residues in animal products such as meat, milk and eggs. These residues, when taken up by humans through consumption of such foods, can enhance resistance of microorganisms to antimicrobials. There is also an inordinate spread of foodborne infections at households, some of which may be resistant, due to cross-contamination of foods particularly vegetables and fruits. Other contributors to foodborne infections in households include poor personal hygiene, improper food handling and preparation, inadequate cooking, and lack of awareness of food safety measures [25]. It is therefore imperative to create awareness on appropriate food hygiene and safety practices to prevent the occurrence of foodborne diseases which will contribute to reduction in the spread of resistant microorganisms in many LMICs.

### Contribution of Environmental Health Practitioners to preventing antimicrobial resistance

Environmental Health Practitioners (EHPs) in LMICs carry out several roles that contribute to the prevention of AMR. As part of their work in WASH, EHPs conduct water sampling and analysis to identify contaminated water sources, and advise community members on water treatment and the safe water chain from source to the point of consumption in order to prevent WASH-related diseases. EHPs are also involved in sanitary inspection of water sources to identify origins of contamination, and work with local leaders, community health workers, and community members to address the identified risks. Through health education, EHPs also increase awareness on sanitation and hygiene practices such as handwashing with clean water and soap at critical times. They also carry out education on other topics such as solid waste and wastewater management, food hygiene and safety, as well as pollution control. They also enforce compliance to public health legislation through the inspection of premises including schools, markets, landing sites, and other institutions to ensure they have sufficient and satisfactory sanitary and other WASH facilities [26]. Through enforcement of legislation by EHPs, nuisances such as open defecation, poor hygiene practices, and improper disposal of solid and liquid waste, which all contribute to the development of AMR, are abated. These various roles of EHPs contribute to the prevention of WASH-related diseases particularly in LMICs which would otherwise lead to the use of antimicrobials hence reducing the occurrence of AMR.

A key role of EHPs is the inspection food and food products, abattoirs and public eating places to guarantee that food sold for human consumption conforms to quality and safety standards, and hygienic requirements [26]. Indeed, EHPs prohibit the manufacture, preparation, storage and sale of food products that are unfit for human consumption. Therefore, they are involved in assessing whether animal products such as meat, milk and eggs are suitable for human consumption especially regarding observance of withdrawal periods on farms in some LMICs. In addition, EHPs ensure medical examination of food handlers to prevent transmission of infections in many LMICs (including those caused by resistant organisms). Through observance of medical examination by food handlers, transmission of food-borne infections such as those caused by *Salmonella, Clostridium perfringes*, and *Campylobacter* are reduced which limits the use of antimicrobials. These roles of EHPs ensure hygiene and safety of food, and prevent the spread of resistant foodborne infections.

EHPs also carry out research related to AMR, contributing to the body of knowledge on the subject. For example, research related to the WASH drivers of AMR is being carried out in Malawi and Uganda as part of the Drivers of Resistance in Uganda and Malawi (DRUM) research consortium [27]. Through research, EHPs are involved in collection, analysis and dissemination of data related to AMR and the environment. Such data not only supports AMR surveillance but also informs decision making at local, national and international levels. With more evidence on the contribution of Environmental Health to the fight against AMR, it is anticipated that more attention will be given to this profession as part of the One Health approach. Indeed, more resources including funding could be allocated by governments in LMICs and donors to address the Environmental Health drivers of AMR including WASH, waste management, as well as food hygiene and safety.

## Conclusion

Environmental Health is crucial in the prevention of infectious diseases including the spread of resistant organisms and antimicrobial residues in LMICs. Improvement in WASH, waste management, as well as food hygiene and safety can significantly contribute to the prevention of AMR. Working with other professionals in One Health including veterinarians, clinicians and laboratory scientists, EHPs have a key role in preventing the occurrence of AMR including through health education and promotion, surveillance, enforcement of legislation, and research.

## Data Availability

Not applicable.

## Abbreviations

AMR: Antimicrobial Resistance
EHP: Environmental Health Practitioner
LMIC: Low- and middle-income country
WASH: Water, sanitation and hygiene
WHO: World Health Organization
